# Differential Expression of *ABC* Transporter Genes in Brain Vessels vs. Peripheral Tissues and Vessels from Human, Mouse and Rat

**DOI:** 10.3390/pharmaceutics15051563

**Published:** 2023-05-22

**Authors:** Wandong Zhang, Qing Yan Liu, Arsalan S. Haqqani, Ziying Liu, Caroline Sodja, Sonia Leclerc, Ewa Baumann, Christie E. Delaney, Eric Brunette, Danica B. Stanimirovic

**Affiliations:** 1 Human Health Therapeutics Research Centre, National Research Council of Canada, Ottawa, ON K1A 0R6, Canada; 2 Scientific Data Mining/Digital Technology Research Centre, National Research Council of Canada, Ottawa, ON K1A 0R6, Canada

**Keywords:** ABC transporters, brain microvessels, peripheral tissues and vessels, gene expression patterns, RNA-seq, Wes^TM^ analysis, across species

## Abstract

Background: ATP-binding cassette (ABC) transporters comprise a superfamily of genes encoding membrane proteins with nucleotide-binding domains (NBD). These transporters, including drug efflux across the blood–brain barrier (BBB), carry a variety of substrates through plasma membranes against substrate gradients, fueled by hydrolyzing ATP. The expression patterns/enrichment of *ABC* transporter genes in brain microvessels compared to peripheral vessels and tissues are largely uncharacterized. Methods: In this study, the expression patterns of *ABC* transporter genes in brain microvessels, peripheral tissues (lung, liver and spleen) and lung vessels were investigated using RNA-seq and Wes^TM^ analyses in three species: human, mouse and rat. Results: The study demonstrated that *ABC* drug efflux transporter genes (including *ABCB1*, *ABCG2*, *ABCC4* and *ABCC5*) were highly expressed in isolated brain microvessels in all three species studied; the expression of *ABCB1*, *ABCG2*, *ABCC1*, *ABCC4* and *ABCC5* was generally higher in rodent brain microvessels compared to those of humans. In contrast, *ABCC2* and *ABCC3* expression was low in brain microvessels, but high in rodent liver and lung vessels. Overall, most *ABC* transporters (with the exception of drug efflux transporters) were enriched in peripheral tissues compared to brain microvessels in humans, while in rodent species, additional *ABC* transporters were found to be enriched in brain microvessels. Conclusions: This study furthers the understanding of species similarities and differences in the expression patterns of *ABC* transporter genes; this is important for translational studies in drug development. In particular, CNS drug delivery and toxicity may vary among species depending on their unique profiles of *ABC* transporter expression in brain microvessels and BBB.

## 1. Introduction

ATP-binding cassette (ABC) transporters comprise a superfamily of transmembrane proteins that are conserved in evolution and found in many species from prokaryotes to humans [1,2,3]. Functional transporter proteins typically contain one or two ATP-binding domains (also known as nucleotide-binding domain (NBD)) and transmembrane (TM) domains. Each TM domain contains six membrane-spanning alpha-helices and determines specificity for substrates [1,2,3]. Eukaryotic ABC transporters are either full transporters containing two NBDs and two TM domains or half transporters with one NBD and one TM domain. The half transporters may be functional as homodimers, heterodimers or multimeric complexes [1,4]. ABC transporters hydrolyze ATP to provide energy to shuttle a variety of substrates through plasma membranes against substrate concentration gradients [3,5]. The superfamily of 51 ABC transporters in humans is divided into seven subfamilies based on similarity in gene structure (half vs. full transporters), order of the domains and sequence homology in the NBD and TM domain (Accessed on 18 May 2023; https://www.genenames.org/cgi-bin/genefamilies/set/417) [1,2,3].

ABC transporters are widely expressed in different tissues and carry out important physiological functions through the transport of metabolites, ions, lipids/cholesterol/steroids, drugs/xenobiotics, antibiotics, toxins and peptides across biological membranes (known functions of ABC transporters are listed in Supplementary Table S1). Notably, ABCB1/MDR-1 (multidrug resistance 1) P-glycoprotein (ABCB1/Pgp), ABCG2/breast cancer resistant protein (ABCG2/BCRP) and ABCC subfamily members/multidrug resistant proteins (ABCC1-5/MRP1-5) play critical roles in drug efflux transport and in forming barrier functions in the human body, including the blood–brain barrier (BBB), blood-cerebrospinal fluid barrier (BCSFB), blood-retinal barrier, blood-testis barrier and placenta barrier [3,6,7,8,9,10]. These barriers protect organs and tissues from blood-borne toxic substances and metabolic waste. However, they can also impede the delivery of therapeutics into ‘protected’ organs and cells; for example, the BBB-expressed ABC transporters actively extrude substrate drugs, making them inaccessible to the targets in the central nervous system (CNS) [3,11,12,13]. The expression and function of ABC transporters are regulated by a spectrum of endogenous and exogenous factors, including nuclear receptors, xenobiotics/drugs and a variety of inflammatory molecules [12]. Long-term administrations of certain xenobiotics/drugs, such as chemotherapeutic and epilepsy drugs, can up-regulate the expression of ABC drug transporters, leading to multidrug-resistant (MDR) phenotypes, particularly in cancer chemotherapy and at the BBB [3,12]. The mutations in *ABC* transporter genes resulting in disease phenotypes have been described previously [2,3] (Table S1).

*ABC* transporter transgenic and gene knockout (KO) animals, including *Abcb1a*/*1b* KO, *Abcg2*/*Bcrp* KO and *Abcc1*/*Mrp1* KO mice [14,15,16,17], have been widely used in studies of drug development and the mechanisms of drug transport. However, it has been observed that therapeutics developed and tested in animal models often display different efficacy and toxicological profiles in clinical studies in humans [18] due, at least partially, to differences in the expression levels of genes encoding ABC transporters among different species. These species differences undermine the translational predictability of animal study data to humans [19]. The current study employs the next-generation sequencing (NGS) approach to compare the levels of *ABC* transporter gene expression in different tissues across species, particularly focusing on *ABC* drug transporter genes in brain vessels compared to peripheral vessels and tissues. This study furthers the understanding of species similarities and differences in the expression patterns of *ABC* transporter genes, which is particularly important for translational considerations from rodents to humans in drug development.

## 2. Materials and Methods

### 2.1. Human and Rodent Tissues

The use of human tissues in this study was approved by the Research Ethics Board of the National Research Council of Canada. Human brain and lung tissue samples from three individuals were used in the study as described previously [20]. Human post-mortem brain frontal cortex tissues were obtained from the Human Brain and Spinal Fluid Resource Center, VAMC, Los Angeles, CA, USA), which is sponsored by NINDS/NIMN, National Multiple Sclerosis Society, VA Greater Los Angeles Healthcare System, and Veterans Health Services and Research Administration, Department of Veteran Affairs. All patients had signed informed consent. Brain samples from three patients, two female (aged 73 and 76 years) and one male (aged 76 years), ethnicity unknown, all deceased from non-brain-related pathologies (cardiomyopathy, coronary artery and obstructive pulmonary disease), were used in this study. Human lung tissues were from the normal adjacent lung tissues of three NSCLC (non-small cell lung cancer) patients undergoing surgical resections. The surgical samples were deposited in the Lung Tumor Bank managed by the CDHA-Capital District Health Authority in Halifax, Nova Scotia, Canada. All patients signed informed consent as per CDHA-RS/2013-271, which allowed their tissues to be archived in the CDHA Lung Tumor Bank for molecular studies. No additional information about the patients could be disclosed. The use of animals (mice and rats) in this study was approved by the Animal Care Committee of the Human Health Therapeutics Research Centre at the National Research Council of Canada. Three mice (C57BL/6 J strain, males, 9 months old) and three Wistar rats (males, 7 to 8 months old) were used in the study. Rodent species (mouse and rat) are the most commonly used in studies of CNS diseases and the BBB. They are also typical toxicology species used for regulatory approvals. These two rodent species were selected for comparative analyses with humans in this study.

### 2.2. Isolation of Microvessels and Capillaries from Tissues

Microvessels (20–300 µm) were isolated from naïve mouse and rat brains and lungs as well as from human brain tissues, as described previously [20,21]. Three animals of each rodent species were used in the study. All human tissues were frozen and stored at −80 °C until vessel isolation. Rodent vessels were isolated from fresh tissues. Human and rodent tissue homogenization and vessel separations were performed on ice using phosphate-buffered saline (PBS) (Wisent, St-Bruno, QC, Canada) containing a protease inhibitor cocktail (Sigma Aldrich, St. Louis, MO, USA), with instruments pre-chilled on ice. Respective tissues were chopped using a razor blade placed in a 5-mL Wheaton Dounce homogenization tube (Fisher Scientific, Hampton, NH, USA), and 5 mL of PBS buffer was added per tube. Tissues were homogenized with 10 strokes of the pestle connected to the Eberbach Con-Torque Homogenizer (Fisher Scientific, Hampton, NH, USA). Homogenized tissues were transferred using gentle suction and filtered through a stack of series of pluriStrainers in a 50-mL conical tube with a connector ring and strainers (pluriSelect, San Diego, CA, USA) in descending order, as follows: connector ring, 300 µm, 100 µm and 20 µm strainers. We would like to point out that the tissue storage (fresh vs. frozen) and processing may have affected the quality of isolated RNA.

### 2.3. RNA Isolation

RNA was extracted from microvessels, lung, liver and spleen tissue homogenates from humans, rats and mice using the RNeasy Plus Mini kit (Qiagen Inc., Toronto, ON, Canada) and NucleoSpin RNA plus kit (Macherey-Nagel GmbH & Co. KG, Dueren, Germany), respectively, according to the manufacturer’s instructions as described previously [20]. Genomic DNA contamination was removed by the Turbo DNA-Free Kit (Life Technologies, Burlington, ON, Canada). RNA quality was assessed using Agilent Bioanalyzer 2100 (Agilent, Santa Clara, CA, USA).

### 2.4. RNA-Seq

RNA-Seq Libraries were generated using the TruSeq strand RNA kit (Illumina, San Diego, CA, USA), as described previously [20]. The RNA-Seq libraries were quantified by Qbit and qPCR according to the Illumina Sequencing Library qPCR Quantification Guide and the quality of the libraries was evaluated on Agilent Bioanalyzer 2100 using the Agilent DNA-100 chip (Santa Clara, CA, USA). RNA-Seq library sequencing was performed using Illumina Next-Seq500. FASTQ file format was processed by trimming the adaptor sequences, filtering low-quality reads (Phred Score ≤ 20) and eliminating short reads (length ≤ 20 bps) using the software package FASTX-toolkit [Accessed on 18 May 2023; http://hannonlab.cshl.edu/fastx_toolkit/]. STAR (Spliced Transcripts Alignment to a Reference) (v2.5.3a) [22] was used for the alignment of the reads to the reference genome and to generate gene-level read counts. RSEM (RNA-Seq by Expectation-Maximization) (version 1.3.3) [23] was used for alignment to generate transcripts per million (TPM) counts. A mouse reference genome (version GRCm38.p6, M24), a human reference genome (version GRCh38.p13, Genecode 33) and corresponding annotations were used as references for RNA-seq data alignment processes. DESeq2 [24] was used for data normalization and differentially expressed gene identification for each pair-wise comparison.

### 2.5. Public Datasets and Analysis

RNA-seq and microarray data in the public domains were obtained to compare/benchmark the data generated from this study for quality and comparability purposes, as described [20]. For RNA-seq data, raw data corresponding to normal lung and brain samples were obtained from the Sequence Read Archive [25] from the Genomics Data Commons [26]. GTEx (Genotype-Tissue Expression) data were processed using GDC (genomic data commons) reference files using the GDC mRNA analysis pipeline (STAR two-pass) [22]. These data were combined with 12 samples analyzed at NRC and processed using DESeq2 [24].

### 2.6. Automated Western Blot Analysis (Wes^TM^)

Human and rodent brain vessel pellets were lysed in Cellytic MT buffer (Sigma-Aldrich, Oakville, ON, Canada) with 1× complete protease inhibitor (Roche Canada, Mississauga, ON, Canada) on ice. The lysates were incubated on ice for 30 min, vortexed, then centrifuged at 21,000× *g* for 10 min in a Sorvall Legend Micro 21R centrifuge. Protein concentrations were determined using the Quantipro BCA (bicinchoninic acid) Assay Kit (Sigma-Aldrich, Oakville, ON, Canada). Wes^TM^ was run using the 12 to 230 kDa separation module (ProteinSimple) and the mouse or rabbit detection module (ProteinSimple Inc., San Jose, CA, USA). Samples (protein at 0.8 mg/mL) were prepared by combining Master Mix with samples at a 1:4 ratio. Samples and Biotinylated Ladder were heated in an Accublock digital dry bath at 95 °C for 5 min. Samples were cooled to room temperature, vortexed and then centrifuged in a Mandel mini microfuge. Biotinylated ladder, samples, primary and secondary antibodies, and luminol were loaded on the plate and Wes^TM^ was run using the standard protocol. The primary antibodies were rabbit anti-P-glycoprotein (Pgp) (Abcam, Toronto, ON, Canada; Cat# Ab170904, lot GR299351-2, dilution 1:50), mouse anti-MRP1 (Novus Biologicals/Bio-Techne Canada, Toronto, ON; Cat# NB400-156, dilution 1:100), rabbit anti-ABCG2 (Biobyrt, Cambridge, UK; Cat# ORB155559, dilution 1:100) and anti-actin-HRP (Sigma-Aldrich, Oakville, ON, Canada; Cat# A3854, dilution 1:100). HRP (horseradish peroxidase)-conjugated anti-mouse or anti-rabbit secondary antibodies from Wes^TM^ detection modules (ProteinSimple Inc., San Jose, CA, USA) were used for detection. Streptavidin-HRP was used to detect the ladder proteins. The Wes^TM^ densitometry software compass measured the chemiluminescence signal during the Wes run and plotted it as chemiluminescence vs. molecular weight. Data for each sample were first normalized to β-actin in the same lane. The level of the protein in human brain microvessels (BMV) was set as one-fold. The fold change of mouse and rat proteins was calculated relative to that of human proteins (means ± SD).

### 2.7. Statistical Analysis

The data were analyzed and compared by one-way ANOVA among multiple groups; this was followed by Tukey’s multiple comparisons tests or unpaired two-tailed Student’s *t*-tests between the two groups. *p* < 0.05 was considered significant.

## 3. Results

### 3.1. RNA-Seq Datasets: Quality, Comparability and Validation

RNA samples were isolated from brain and peripheral vessels and tissues. Due to limited availability, only cerebral microvessels and lungs were analyzed from humans, whereas cerebral and lung vessels, liver and spleen were analyzed from mice and rats. The quality of the cerebral vessel preparation was confirmed by the expression of brain vessel marker proteins, including TfR (transferrin receptor), IGF1R (insulin-like growth factor receptor-1), CD31/PECAM-1 (Platelet endothelial cell adhesion molecule-1), GFAP (glial fibrillar acid protein) and Collagen IV using immunofluorescence analyses, as described previously [20]. RNA-seq libraries were then generated and sequenced.

To confirm the comparability among datasets, it was essential to evaluate the quality of the RNA-seq datasets generated in this study with similar RNA-seq datasets in the public domain. The analysis showed that RNA-seq datasets generated from total human brains and lungs were highly correlated to RNA-seq datasets in public domains concerning the same tissues, with a correlation co-efficient of 0.96 [20]. The comparability of different datasets generated in our own studies was also analyzed, giving a correlation co-efficient between 0.94 and 0.97. These analyses confirmed the quality and comparability of the datasets generated in the study, as demonstrated in our previous report [20], mitigating the impact of low sample numbers.

### 3.2. The Expression of ABC Transporter Genes in Brain Vessels across Species

**Figure 1:**
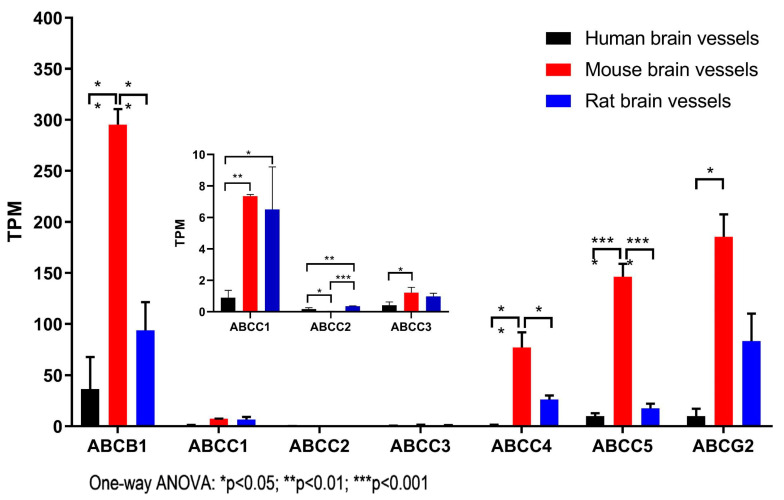
The expression of *ABC* drug transporter genes in brain microvessels of humans, mice and rats analyzed by transcript per million (TPM) across species.

The first objective of this study was to compare the expression of *ABC* transporter genes in isolated brain vessels from different species to understand potential species differences in BBB function in xenobiotic and lipid transport. The brain microvessels analyzed in this study were highly enriched in brain endothelial cells (BEC), but also contained pericytes and astrocyte end-feet, as described previously [20]. It is important to note that a very high expression level of a gene in ‘contaminating’ cells coupled with low or absent expression in a dominant cell type tissue could result in misinterpretation of data obtained from mixed cell tissue. While this is a confounding factor in this study, it has been shown previously that transporters expressed in both BEC and other cells of the neurovascular unit contribute to drug influx and efflux across the BBB [27]. Therefore, conclusions concerning the impact of *ABC* transporter gene expressions in microvessel preparations on the BBB function could be drawn. To compare the levels of gene expression across species, the normalized read counts of RNA-seq data were converted to transcripts per million (TPM) counts [20]. The levels of *ABC* transporter gene expression were then compared across humans, mice, and rats based on TPM data analysis, and the results are presented in Table 1. The data on the major *ABC* drug efflux transporter genes were extracted from Table 1, analyzed and presented in Figure 1. ABCB1/MDR1/P-glycoprotein (Pgp) is a drug efflux transporter responsible for multidrug resistance in cancer chemotherapy and drug efflux at the BBB. There is one *ABCB1* gene in the human genome but there are two isoforms, *Abcb1a* and *Abcb1b*, in that of rodents. The TPM values of *Abcb1a* and *Abcb1b* RNA-seq data were combined and then compared to human *ABCB1* (Table 1; Figure 1). It was noted that the level of *Abcb1b* expression was lower compared to that of *Abcb1a* in mouse and rat cerebral vessels, suggesting that *Abcb1a* is the main isoform of a drug efflux transporter in cerebral vessels in rodents (Table 1). It was observed that *Abcb1* expression levels in the cerebral vessels of mice (295.34 ± 26.58) were three-fold and eightfold higher than those in rats (93.78 ± 47.90) and humans (36.62 ± 54) (Figure 1; Table 1) (one-way ANOVA, ** *p* < 0.01), respectively. The expression levels of *ABCB1* in human brain vessels were significantly lower compared to those in either rat or mouse brain vessels.

ABCC1/MRP1 has been identified and characterized as one of the major drug efflux transporters in multidrug resistance regarding cancer chemotherapy [3,6]. In this study, the expression levels of the *ABCC1*/*MRP1*, *ABCC2*/*MRP2* and *ABCC3*/*MRP3* genes were found to be generally low in cerebral vessels in all three species examined (Figure 1 insert), although some differences were observed; for example, *Abcc2* in rat cerebral vessels (0.35 ± 0.05) was slightly higher than that in mouse cerebral vessels (0.03 ± 0.01) (one-way ANOVA, ** *p* < 0.01) (Figure 1; Table 1). Interestingly, the expression levels of the *ABCC4*/*MRP4* and *ABCC5*/*MRP5* genes were significantly higher than those of *ABCC1-3* in brain vessels in all three species studied (Table 1; Figure 1). Similar to *ABCB1*, the expression levels of both *ABCC4*/*MRP4* and *ABCC5*/*MRP5* were significantly higher in the cerebral vessels of mice compared to those in humans or rats (Figure 1; Table 1). These results suggest that *ABCC4*/*MRP-4* and *ABCC5*/*MRP-5* may be more important than *ABCC1-3*/*MRP1-3* for the transport function of brain vessels.

ABCG2/BCRP is a half-transporter in the ABCG subfamily and can form homo- or hetero-dimers; its substrate spectrum mostly overlaps with that of ABCB1 [1,2,3,6,28]. ABCG2/BCRP may be complementary to ABCB1 and is critical for BBB transport [1,2,3,6,28]. The RNA-seq data in this study found that *ABCG2*/*BCRP* expression was higher than *ABCC1-5*/*MRP1-5*, but lower than *ABCB1* in brain vessels across studied species (Table 1; Figure 1), with the expression levels in mouse brain vessels (185.35 ± 38.42) higher than those in either humans (10.01 ± 12.63) (* *p* < 0.05) or rats (83.22 ± 46.64) (Figure 1A; Table 1). Overall, the expression levels of *ABC* drug transporter genes in cerebral vessels were higher in rodents than in humans (mice > rats > human), with *ABCB1*, *ABCG2*, *ABCC4* and *ABCC5* being highly expressed or enriched in cerebral vessels.

The protein expression of three major drug efflux transporters, including ABCB1/Pgp, ABCC1/MRP-1 and ABCG2/BCRP, was then validated in human, mouse and rat cerebral vessels by quantitative Wes^TM^ analysis using species cross-reactive antibodies (Supplementary Figure S1A,B). Protein-level expression analyses showed some discrepancies from the RNA-seq analyses. For example, ABCB1/Pgp protein levels were higher in rat cerebral vessels compared to those in mouse cerebral vessels (Supplementary Figure S1A). The Wes^TM^ analysis results were consistent with the RNA-seq analysis results for *ABCC1/MRP1* and *ABCG2/BCRP* (Supplementary Figure S1), showing the order of protein expression abundance among species similar to what was observed at the mRNA level.

**Figure 2:**
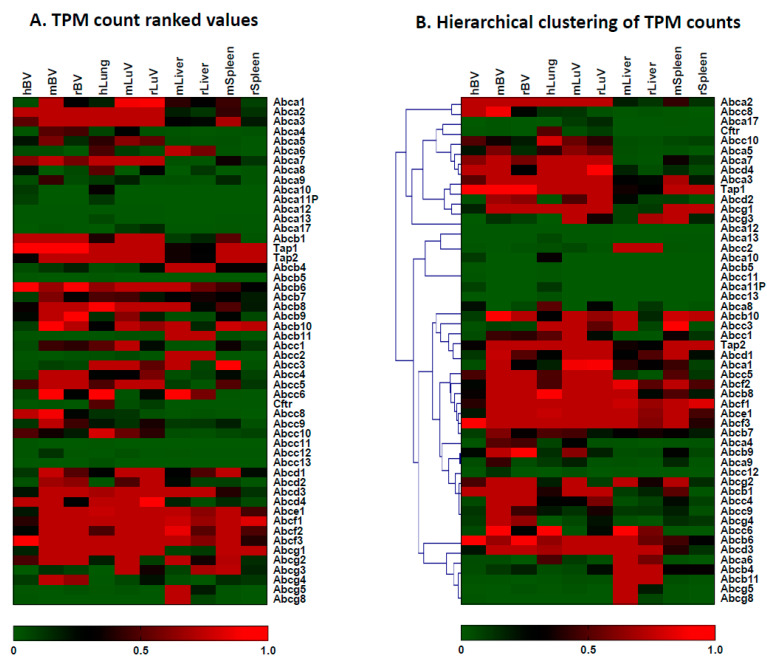
Heatmap for the levels of *ABC* transporter gene expression in humans, mice and rats (h for humans; m for mice and r for rats in the graphs): Three samples were used in RNA-seq analyses for cerebral vessels and peripheral tissues and lung vessels from humans, mice and rats. The RNA-seq data were converted to transcript per million (TPM) counts for comparison across species. The means of three samples (TPM counts) were used in the following analysis. (**A**). Transcript per million counts (TPM) ranking for *ABC* transporter gene expression in human, mouse and rat brain vessels vs. peripheral tissues and lung vessels. (**B**). TPM ranking values of hierarchical clustering for *ABC* transporter gene expression in human, mouse and rat brain vessels vs. peripheral tissues and lung vessels.

ABCA subfamily transporters, composed of 13 members in humans and 17 members in rodents (Table 1), are mostly involved in lipid and cholesterol transport. ABCA1 is a well-characterized cholesterol transporter that mediates cellular cholesterol efflux in the brain and influences neuroinflammation and neurodegeneration [29,30]. The expression of *ABCA1* was significantly higher in mouse brain vessels (19.45 ± 1.72) compared to rat (5.90 ± 1.47; *** *p* < 0.001) or human brain vessels (0.69 ± 0.66; ** *p* < 0.01) (Table 1). The expression of *ABCA1* in rat brain vessels was also significantly higher than that in human brain vessels (*** *p* < 0.001) (Table 1). *ABCA2* and *A3* were found to be highly expressed in cerebral vessels across species (Table 1; Figure 2). The level of *ABCA2* in human cerebral vessels (208.09 ± 70.27) was higher than that in either rat (71.63 ± 25.12; one-way ANOVA, * *p* = 0.0217) or mouse (107.57 ± 15.42) cerebral vessels; while the level of *ABCA3* was significantly lower in humans (10.85 ± 6.45) compared to either mice (35.98 ± 2.22; ** *p* = 0.005) or rats (26.49 ± 7.73; * *p* = 0.0416) (one-way ANOVA) (Table 1). ABCA2 is a lipid transporter involved in the maintenance of homeostasis of cholesterol and sphingolipids and is highly expressed in brain tissue [31]. *ABCA2* knockout mice display developmental defects similar to aberrant myelination [31]. ABCA3 is involved in phospholipid transport for surfactant production in lung tissue [32] and is overexpressed in childhood acute myeloid leukemia [33]. Nevertheless, the role of ABCA3 in cerebral vessels is still poorly understood. Both ABCA7 and ABCA8 are involved in the transport of cholesterol or/and phospholipids and play roles in sphingomyelin synthesis and the generation of HDL-like particles [34,35,36]. The two transporter genes were moderately expressed in cerebral vessels across species (Table 1; Figure 2), although the levels of *ABCA8* expression were higher in mice (13.82 ± 3.03) and rats (13.52 ± 2.97) than in humans (4.84 ± 1.54; one-way ANOVA, * *p* < 0.05) (Table 1). The roles of ABCA7 variants in sterol and lipid transport and metabolism makes them one of the top risk factors for Alzheimer’s disease development [36,37,38,39,40].

There are 11 members of the ABCB subfamily. In addition to the drug efflux transporter ABCB1/Pgp, the other members have diverse or unclear functions. ABCB2/TAP1 (Transporter associated with antigen processing 1) and ABCB3/TAP2 are involved in peptide transport or peptide antigen presentation for immune response [3,6]. These two genes were expressed at moderate to high levels in cerebral vessels across the species (Table 1; Figure 2). The same is true for *ABCB6* (Table 1; Figure 2) which encodes a Fe/S cluster transporter for mitochondrion [6], as well as for ABCB8 and B9, which are both involved in the maintenance of mitochondrial iron homeostasis, maturation of cytosolic Fe/S proteins and transport of peptides and phospholipids [6,41]. ABCB9 is a TAP-like half-transporter associated with lysosomes and may function as a peptide translocase and phosphatidylserine floppase [42,43]. The levels of both *ABCB8* and *ABCB9* expression were significantly lower in human cerebral vessels compared to mouse or rat cerebral vessels (Table 1; Figure 2).

There are 13 members of the ABCC subfamily. In addition to *ABCC1-5*, *ABCC8* was similarly expressed at moderate to low levels in cerebral vessels across species (Table 1; Figure 2). Interestingly, *ABCC6* and *C9* were expressed at moderate levels in mouse cerebral vessels (*ABCC6*: 15.72 ± 4.27; *ABCC9*: 26.48 ± 10.26) but significantly lower in human (*ABCC6*: 1.04 ± 0.74, ** *p* < 0.01; *ABCC9*: 2.40 ± 1.95, ** *p* < 0.01) and rat (*ABCC6*: 4.69 ± 3.00, ** *p* < 0.01; *ABCC9*: 8.14 ± 2.74, * *p* < 0.05) cerebral vessels (one-way ANOVA) (Table 1; Figure 2). ABCC6 is a GS-X pump involved in the transport of glutathione and peptides, while ABCC8 and C9 are known as sulfonylurea receptors and regulate ATP-sensitive K+ channels [6,44] (Table S1).

There are four, one and three members of the ABCD, ABCE and ABCF subfamilies, respectively, in all three species (Table 1). All the members were expressed at low levels in human cerebral vessels except *ABCD4* and *ABCF3* (Table 1; Figure 2). However, all the members were expressed at moderate to relatively high levels in mouse and rat cerebral vessels (Table 1; Figure 2). The four *ABCD* subfamily members are half-transporters and may be involved in peroxisome-related functions [2,6]. The functions of ABCE and ABCF1-3 are not well understood.

ABCG subfamily members, except for ABCG2 and Abcg3, are involved in sterol or/and phospholipid transport [1,2,3,6,45,46]. *ABCG1* and *G4* were expressed at moderate levels in rodent cerebral vessels but at lower levels in human cerebral vessels (Table 1; Figure 2). Both genes are known to be highly expressed in human brain tissue [3,6] and their function in brain vessels may involve the transport of sterol and phospholipids across the BBB.

### 3.3. The Expression of ABC Transporter Genes in Brain Vessels vs. Lung Vessels, Liver and Spleen

**Figure 3:**
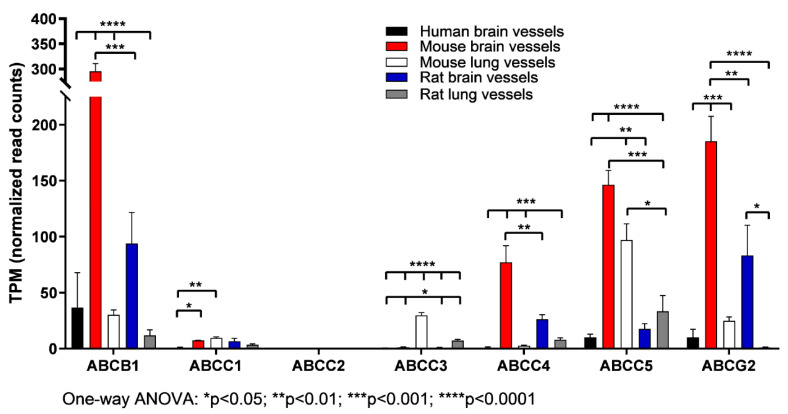
Expression of *ABC* drug transporter genes in cerebral and lung vessels across species. Three samples of cerebral vessels from humans, mice and rats were used in RNA-seq analysis. The RNA-seq data were then converted to transcript per million (TPM) counts for comparison across species. Mean ± SD from three samples was used in the analysis (Table 1, Table 2, Table 3 and Table 4). One-way ANOVA was used for statistical analysis (* *p* < 0.05; ** *p* < 0.01; *** *p* < 0.001; **** *p* < 0.0001).

The second and broader objective of the study was to evaluate the relative enrichment of *ABC* transporter gene expression in brain vessels vs. lung vessels, lung, liver and spleen tissues. As shown in Figure 2 and Table 1, Table 2, Table 3 and Table 4, the expression levels of *ABC* transporter genes and their distribution varied among different tissues and vessels across species. Liver, lung and spleen tissues used in the study are a mixture of different cell types; therefore, the expression data shown reflect tissue-averaged levels of all cell types present in that particular tissue. For example, *ABCB1*, *ABCG2* and *ABCC5*/*MRP-5* were expressed at high levels in cerebral vessels across the species compared with lung vessels (Figure 3), while *ABCC4*/*MRP-4* and *ABCC9* were highly expressed in mouse and rat cerebral vessels but not in human cerebral vessels. *ABCC8*, which encodes an ATP-sensitive K^+^ channel, was highly expressed in human cerebral vessels but not in other human, mouse and rat tissues analyzed in this study (Figure 2 and Figure 3; Table 1, Table 2, Table 3 and Table 4). *ABCC8* is typically expressed in pancreatic insulin-secreting cells and pituitary glands (Table S1). In addition to their expression in the liver, *ABCA2*/*A3* and *ABCB6*/*B8*/*B9* were expressed at moderate to high levels in brain vessels (Figure 2; Table 1, Table 2, Table 3 and Table 4). A moderate level of *ABCA2* and a high level of *ABCA3* were expressed in lung tissues and lung vessels (Table 2, Table 3 and Table 4). *ABCC1*/*MRP1* was moderately expressed in human and mouse lung vessels/tissues but not in other tissues analyzed in this study, while *ABCC2/MRP-2* was highly expressed in mouse and rat liver tissues. *ABCC3/MRP3* was expressed at moderate to low levels in mouse and rat cerebral vessels as well as in human and mouse lung tissues/vessels and mouse liver and spleen, but not in other tissues across the species (Figure 2 and Figure 3; Table 1, Table 2, Table 3 and Table 4). *ABCC5/MRP5* was expressed at moderate to high levels in lung tissues/vessels across the species (Figure 2 and Figure 3). These results suggest that drug transporters ABCB1, ABCG2 and ABCC5/MRP5 appear to be important in cerebral vessels across species, whereas the lower expression of ABCC4/MRP4 in human vessels compared to that in mouse and rat vessels suggests its higher functional importance in rodent species (Table S1). Drug transporters ABCC1/MRP-1, ABCC2/MRP-2 and ABCC3/MRP-3 play a dominant role in the liver and lungs (Table S1), while they appear to be functionally less important in brain vessels.

**Figure 4:**
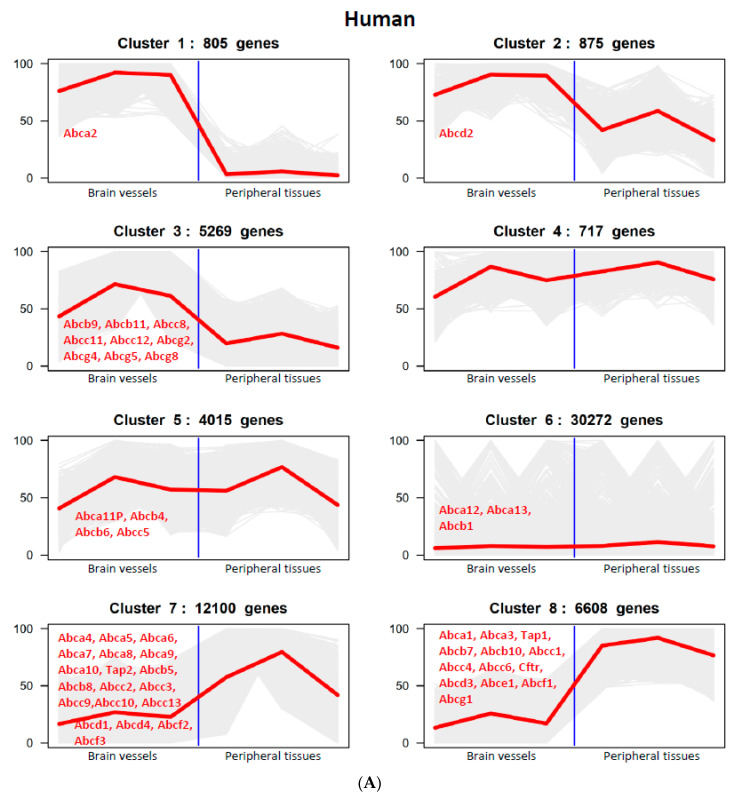

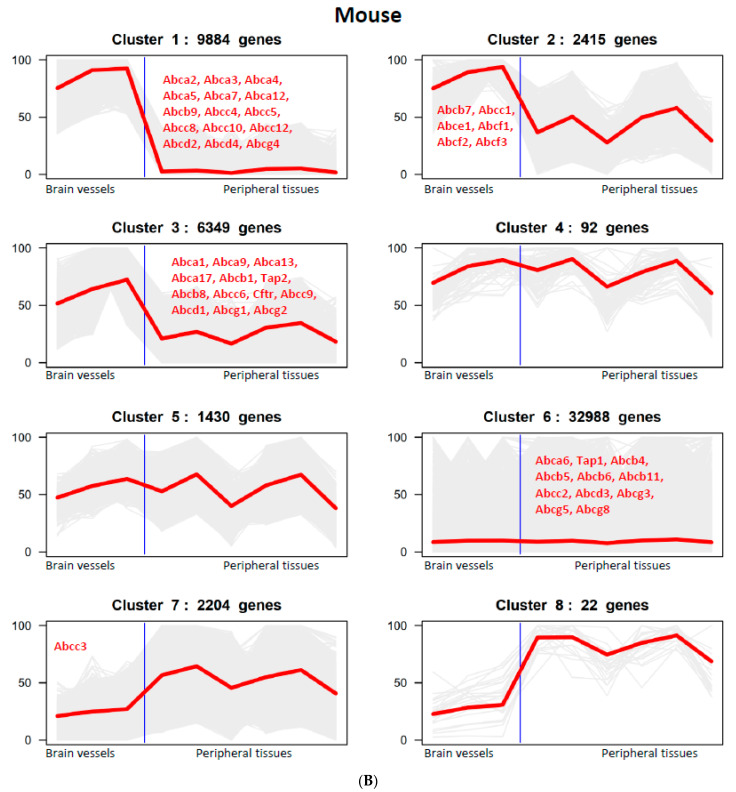

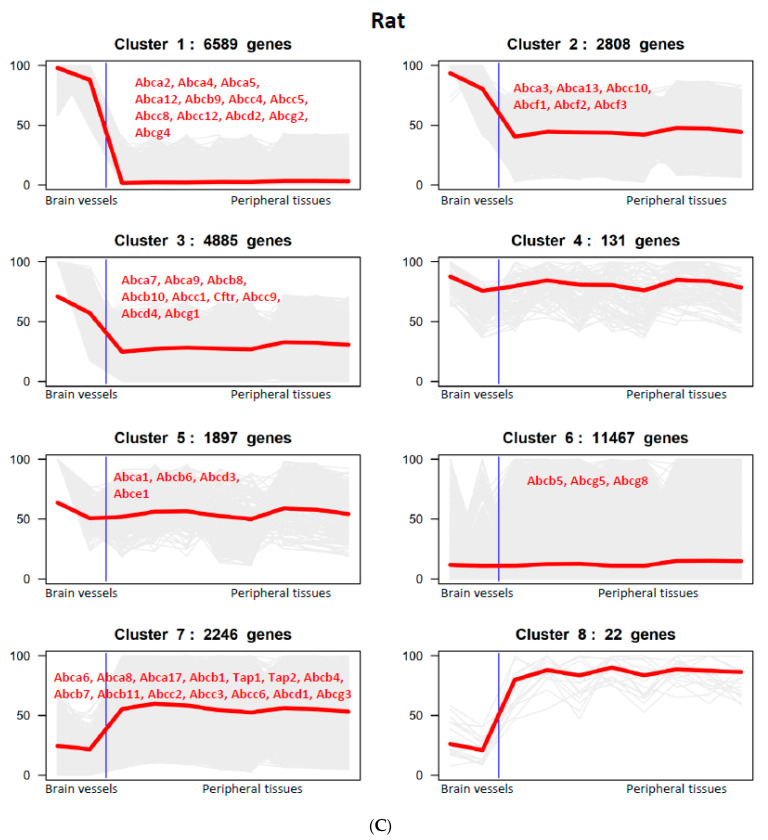
K-means clustering of RNA-seq data (TPM counts) showing classification of *ABC* transporter gene expression in brain vessels vs. peripheral tissues. (**A**). Human brain vessels vs. human lung tissues. (**B**). Mouse brain vessels vs. mouse peripheral tissues (including mouse liver and spleen tissues). (**C**). Rat brain vessels vs. rat peripheral tissues (including rat liver and spleen tissues). The 8 clusters in each species correspond to the following: (1) high in brain vessels and low in peripheral tissues, (2) high in brain vessels and medium in peripheral tissues, (3) medium in brain vessels and low in peripheral tissues, (4) high in brain vessels and high in peripheral tissues, (5) medium in brain vessels and medium in peripheral tissues, (6) low in brain vessels and low in peripheral tissues, (7) low in brain vessels and medium in peripheral tissues, (8) low in brain vessels and high in peripheral tissues.

K-means clustering analysis was performed on human, mouse and rat RNA-seq data (TPM counts). Based on the abundance of gene expression, eight clusters of genes were identified from the analysis of brain vessel vs. peripheral tissue expression data (including liver and spleen) (Figure 4). The analysis considered *ABC* transporter gene expression relative to the expression of all the genes detected in the RNA-seq dataset of each species. Cluster 1 shows the *ABC* transporter genes that were expressed at very high levels in brain vessels but at very low levels in peripheral tissues (Figure 4). *ABCA2* was the only gene found among 805 genes in humans (Figure 4A). There were 15 *ABC* transporter genes (among 9884 genes in cluster 1) in mice (Figure 4B) and 12 *ABC* transporter genes in rats (among 6589 genes in cluster 1) (Figure 4C) that showed this profile. Cluster 2 represents the *ABC* transporter genes that were expressed at very high levels in brain vessels but at moderate levels in peripheral tissues. *ABCD2* was the only gene found among 875 genes in humans in cluster 2. There were 6 *ABC* transporter genes in both mice (among 2415 genes) (Figure 4B) and rats (2808 genes) (Figure 4C) in cluster 2. Cluster 3 includes genes that were expressed at high levels in brain vessels but at moderate to low levels in peripheral tissues. There were 9 *ABC* transporter genes in humans (among 5269 genes in cluster 3) (Figure 4A), 13 *ABC* transporter genes in mice (among 6349 genes) (Figure 4B) and 11 *ABC* transporter genes in rats (among 4885 genes in cluster 3) (Figure 4C). Cluster 4 identifies genes that were highly expressed in both brain vessels and peripheral tissues; none such *ABC* transporter gene was identified in any of the studied species (Figure 4A–C). Cluster 5 includes the genes that were expressed moderately in both brain vessels and peripheral tissues. There were 4 *ABC* transporter genes in both humans (among 4015 genes) (Figure 4A) and rats (among 1897 genes) and none in mice (among 1430 genes) in cluster 5 (Figure 4B). Cluster 6 identifies *ABC* transporter genes that were expressed at low levels in both brain vessels and peripheral tissues (Figure 4). Cluster 7 includes *ABC* transporter genes that were expressed at low levels in brain vessels but moderate to high levels in peripheral tissues. Cluster 8 identifies *ABC* transporter genes that were expressed at low levels in brain vessels but at very high levels in peripheral tissues, as shown in Figure 4. The clustering analysis shows *ABC* transporter gene expression patterns relative to all genes expressed in brain vessels vs. those expressed in peripheral tissues in each of the species. The patterns of *ABC* transporter gene clustering in these subgroups were similar in mice and rats.

Finally, the data generated allowed us to analyze tissue-specific enrichment of *ABC* genes and compare this across preclinical species and humans. Clusters 1 and 8 represent the *ABC* transporter genes that were enriched in either brain vessels or peripheral tissues, respectively. Species differences between humans and rodents in *ABC* transporter gene enrichment were clearly identified through clustering analyses. There were over 10 *ABC* transporter genes enriched in rodent brain vessels but only one in human brain vessels (Figure 4). The opposite was true for the *ABC* transporter genes enriched in peripheral tissues; 11 *ABC* transporter genes were enriched in human peripheral (lung) tissues, but none were enriched in rodent peripheral (liver and spleen) tissues (Figure 4).

Species differences were also found regarding the number of *ABC* transporter genes encoded in human and rodent genomes. In addition to the well-known presence of two isoforms of *Abcb1* (*Abcb1a* and *Abcb1b*) in rodents compared to one *ABCB1* in humans, other species differences were also identified. For *ABCA* subfamily genes, four additional genes (*Abca14* to *Abca17*) were present in the rodent but not in the human genome. However, these four genes were expressed at extremely low levels in both rodent brain vessels and rodent tissues analyzed in our study (Table 2, Table 3 and Table 4). *Abcg3* was found in rodents but not in humans (Table 2, Table 3 and Table 4). Bioinformatics analysis suggested that *Abcg3* is closely related to *Abcg2* and was predicted to be a drug efflux transporter, although its function has not been characterized [2,47].

## 4. Discussion

ABC transporters play critical roles in transporting a variety of substrates across plasma membranes against substrate gradients, although the function of some of them is still not completely understood [1,2,3]. This study analyzed the expression and distribution patterns of *ABC* transporter genes and demonstrated marked differences in their tissue abundance, as well as differential expression patterns in three studied species—human, mouse and rat. In particular, the study identified species differences in *ABC* transporter enrichment and expression levels in brain vessels, relevant for the function of BBB.

Several prior studies investigated and quantified the expression of transporters [including some ABC and solute carrier (SLC) transporters] in cerebral vessels and the liver or kidneys of humans, mice, rats and non-human primates using proteomics approaches [19,48,49,50,51,52]. However, none of these studies analyzed the expression patterns of all known *ABC* transporter genes in brain vessels vs. peripheral (lung, liver and spleen) tissues in different species. One of the most important features of cerebral vessels is the cellular and molecular anatomy of brain endothelial and adjacent cells that underlies the BBB. The BBB restricts the passage of blood-borne neurotoxic substances, xenobiotics and drugs into the brain and maintains the homeostasis of the CNS. In addition to physical tightness for molecules > 500 D, the drug transport barrier is achieved mostly by the polarized expression of ABC drug efflux transporters such as ABCB1/Pgp, ABCG2/BCRP and ABCC4/5/MRP4/5 [3,6,53]. RNA-seq analyses performed in this study revealed that *Abcb1a*, *Abcg2*, *Abcc4* or/and *Abcc5* were highly expressed in the brain vessels of mice and rats compared to other *ABC* transporter genes, as well as highly enriched compared to the peripheral tissues analyzed. Similar to mice and rats, *ABCB1*, *ABCG2* and *ABCC5* were also highly abundant and enriched in human cerebral vessels compared to lung tissue.

To complement the RNA-Seq analyses, the protein level expression of major drug efflux transporters ABCB1/Pgp, ABCC1/MRP1 and ABCG2/BCRP was confirmed in the isolated cerebral vessels from humans, mice and rats, showing general congruency between gene and protein expression, with some variations.

It is important to note that the expression levels of *ABC* transporter genes were different in the same tissues between humans and rodents. For example, the expression levels of *ABCB1* and *ABCG2* were significantly higher in mouse and rat brain vessels than that in human brain vessels. Given that both ABCB1 and ABCG2 are major drug efflux transporters expressed at the BBB and additional transporter genes are present in rodents (such as Abcb1b), there are different levels of gene expressions/abundance in brain vessels, and since the high sensitivity of humans to drug toxicity, the direct extrapolation of drug testing/transport assays in animal in vitro and in vivo BBB models should be cautious.

Furthermore, *Abcc4*/*Mrp4* and *Abcc5*/*Mrp5* were also found to be more abundantly expressed in rodent brain vessels compared to human brain vessels. Hence, the above consideration of ‘translatability’ should also apply to these GS-X pump drug efflux transporters and their substrate spectrums, which are different from those of ABCB1 and ABCG2.

In addition to *ABCB1*, *ABCG2* and *ABCC5* drug transporter genes, there was only one additional *ABC* transporter gene (*ABCA2*) that was highly enriched/expressed in human brain vessels. In contrast, clustering analyses identified 15 and 12 other *ABC* genes in mice and rats, respectively, that were highly enriched/expressed in brain vessels; the identified genes mostly overlapped between rodent species.

Generally, higher expression levels of these drug transporters were observed in rodent cerebral vessels compared to human cerebral vessels, consistent with previous reports in the literature [48,54,55]. The evolutionary adaptation to the higher exposure to broader sources of foods and environmental toxins in rodents compared to humans has been considered as a potential explanation for this ‘enhanced’ barrier function through the induced expression of drug efflux transporters. The neurotoxic substances or xenobiotics in the food sources of rodents might induce higher habitual levels of gene expressions [8,12]. Alternatively, genetic changes might have occurred, for example, in regulatory regions of drug transporter genes, during evolution, which led to higher-level expression (or silencing) in different species. For example, there are two isoforms of Abcb1/Pgp in both rodent species and only one isoform of ABCB1/Pgp in humans. The two rodent isoforms may complement each other functionally to achieve a wider substrate spectrum than that of humans. For example, one study found that bisphenol A is likely a substrate for rat Abcb1b/mdr1b based on high ATPase activity assay, but not for human ABCB1/MDR1 or rat Abcb1a/mdr1a [56]. The homology between human ABCB1 and rodent Abcb1a protein sequences is approximately 87%. The sequence difference may determine the affinity of drug substrates to the transporter protein, the spectrum of substrates accepted by the transporter and their transporting efficiency. One study found that the substrate recognition or transport efficiency of ABCB1/Pgp differs between humans and mice for certain antiepileptic drugs [57]. Strong drug efflux efficiency or a wider substrate spectrum ensures that the blood-borne neurotoxic agents and xenobiotics are not accessible to the CNS [55,56,57]. Similarly, *Abcg3*, predicted to be a drug transporter gene and considered to be closely related to *Abcg2* [47], is present in rodents but not in humans.

Neuro-drug development and testing often employ in vitro and in vivo rodent BBB models. Mice and rats are the most used pre-clinical species for CNS disease models and toxicology studies. It has been observed that many drug candidates found to be efficacious and safe in rodent models have failed in human clinical trials [58,59]. It appears that humans might be more sensitive or have stronger toxic responses to drugs than rodents [60], which might be due to differences between human and rodent drug transporter expression levels or the presence/absence of variant genes. The differences between humans and rodents have been noted in preclinical studies [58,59,60], and the results obtained from rodent in vitro and in vivo models may not be completely translatable to human models [58,59,60].

In addition to the enrichment of *ABCB1*/*Abcb1a* in cerebral vessels, this study also found that *ABCA2* was highly expressed in brain vessels relative to other tissues and other *ABC* transporter genes in both rodent and human species. It was observed that *ABCA2* expression in human brain vessels was higher than that in rodent brain vessels. ABCA2 is a transporter for cholesterol and sphingolipids [31] and is involved in supplying cholesterol-rich brains with essential lipids. *Abca2* knockout in mice resulted in developmental defects, suggesting that ABCA2 is a transporter with more specific roles in brain vessels and high functional significance to brain development [31]. The expression levels of *ABCA1*, *ABCG1* and *ABCG4* cholesterol/lipid transporter genes in the brain [29,30,45,46] were higher in rodent brain vessels than in human brain vessels.

This study provided additional evidence that some *ABC* transporter genes are specifically expressed or enriched in certain tissues and vessels, such as, for example, the liver and lungs. Both *ABCC2*/*MRP2* and *ABCC3*/*MRP3*, known to encode GS-X pump drug efflux transporters [1,2,3,6], were highly expressed in the liver; *ABCC2*/*MRP2* expression in rodent liver was comparable to or higher than that of *ABCB1* in brain vessels. Another liver-specific transporter detected in the study was *Abcb11*, which mediates bile acid export.

In contrast, *ABCC1*/*MRP1*, *ABCC3*/*MRP3* and *ABCC5*/*MRP5* were found to be expressed at higher levels in human and rodent lung vessels relative to their expression in brain vessels. ABCC4 and ABCC5, both transporters involved in the cellular export of cyclic nucleotides such as cAMP and cGMP, may contribute to the elimination pathway for cyclic nucleotides in the regulation of signal transduction [2]. Higher levels of *ABCC4*/*MRP4* and *ABCC5*/*MRP5* in rodent lung vessels, as well as higher levels of *ABCC1*/*MRP1* and *ABCC3*/*MRP3* in human lungs, suggest that the functions of these transporters are needed for essential lung function. The high expression of *ABCC4* in airway epithelia and its role in the regulation of CFTR/ABCC7 ion channel activity have been documented [61]. ABCA3 is involved in phospholipid transport and implicated in surfactant production in the lungs [6,32]. *ABCA3* was also moderately expressed in rodent brain vessels, although its functional role in brain vessels remains unclear.

The limitations of this study include the use of three human brain samples of older donors (>73 years, two females and one male) with underlying non-brain diseases likely requiring medications, some of which may had altered the expression of *ABC* transporter genes. In addition, there was a scarcity of clinical information available for the three patients’ lung tissues. It is noted that the age, gender, underlying diseases, medications and ethnicities of the patients could have impacted *ABC* transporter expression. Human brain and lung tissues were frozen and stored at −80 °C before vessel isolation and RNA extraction, while mouse and rodent vessels and RNA were isolated from fresh tissues. Moreover, adult male mice and rats were used; gender/sex hormones may have affected *ABC* transporter expression [62]. Given that the limitations, as well as the sample size, selection and preparation, may have impacted the *ABC* transporter gene expression observed in the datasets, generalization of the findings observed in this study should be cautioned and made within the context of this particular study. Some of these deficiencies were mitigated by the extensive comparisons and high correlative alignment of the generated datasets with those available in public repositories. Although multicellular tissues were used for sequencing, the impact of the overall tissue expression of *ABC* transporter genes in different species on drug biodistribution and potential tissue toxicity could be inferred from the dataset. Furthermore, many of the transporters analyzed in this study were less studied in the literature, providing useful insights about their potential functional importance in specific tissues. Since gene expression at the mRNA level may not be completely translated at the protein level, future investigations on species differences at the protein levels of ABC transporters (such as proteomics analysis) and even at the functional levels (such as drug pharmacokinetics) are warranted.

## 5. Conclusions

In summary, our study provides the first overview of *ABC* transporter gene expression patterns in brain vessels vs. peripheral tissues (lung, liver and spleen) and lung vessels across species. RNA-seq and Wes^TM^ analyses on brain vessels demonstrated that *ABC* drug transporter genes, including *ABCB1*, *ABCG2*/*BCRP*, *ABCC4*/*MRP4* and *ABCC5*/*MRP5*, were highly expressed in both human and rodent brain vessels. These transporters cover a wide spectrum of drug substrates, preventing blood-borne neurotoxic substances from entering the CNS. It is noted that the expression levels of *ABC* drug transporter genes were generally higher in rodent cerebral vessels compared to human cerebral vessels. Some of the other *ABC* transporter genes were expressed more abundantly or specifically in peripheral organs, such as the liver and lungs. In each case, there were significant differences in patterns of expression or relative enrichment between rodents and humans. Therefore, the interpretation (and translation) of drug biodistribution and toxicity studies with substrates of these transporters performed in rodents must take into account differences in the abundance and patterns of expression of ABC transporters in both peripheral tissues and brain vessels with specialized barriers, such as the BBB.

## Figures and Tables

**Table 1:** The levels of *ABC* transporter gene expression in cerebral vessels of humans, mice and rats (RNA-seq analysis by transcripts per million counts (TPM)) ^§^.

ABC Transporter Genes	Human (Mean ± SD)	Mouse (Mean ± SD)	Rat (Mean ± SD)
*ABCA1*	0.69 ± 0.66	19.45 ± 1.72 ^h^**; ^r^***	5.90 ± 1.47 ^h^***
*ABCA2*	208.09 ± 70.27 ^r^*	107.57 ± 15.42	71.63 ± 25.12
*ABCA3*	10.85 ± 6.45	35.98 ± 2.22 ^h^**	26.49 ± 7.73 ^h^*
*ABCA4*	0.19 ± 0.15	8.22 ± 1.19 ^h^*	7.85 ± 4.64 ^h^*
*ABCA5*	4.51 ± 1.34	9.77 ± 1.16 ^r^***; ^h^**	2.62 ± 0.72
*ABCA6*	0.88 ± 0.08 ^m^*; ^r^****	0.67 ± 0.10 ^r^***	0.03 ± 0.01
*ABCA7*	13.04 ± 3.68	19.29 ± 1.78	9.86 ± 4.96
*ABCA8*	4.84 ± 1.54	13.82 ± 3.03 ^h^*	13.52 ± 2.97 ^h^*
*ABCA9*	0.81 ± 0.91	7.12 ± 1.23 ^r^***; ^h^***	0.78 ± 0.26
*ABCA10*	1.93 ± 1.26	0.00 ± 0.00	0.00 ± 0.00
*ABCA12*	0.01 ± 0.00	0.03 ± 0.01	0.05 ± 0.02
*ABCA13*	0.02 ± 0.01	0.03 ± 0.01	0.05 ± 0.02
*Abca14*	Not present	0.00 ± 0.01	0.00 ± 0.00
*Abca15*	Not present	0.00 ± 0.00	0.00 ± 0.00
*Abca16*	Not present	0.00 ± 0.00	0.04 ± 0.04
*Abca17*	Not present	0.20 ± 0.13	0.04 ± 0.02
*Abcb1a*	Homologous to ABCB1	290.67 ± 26.70 ^r^##	93.50 ± 47.78
*Abcb1b*	Homologous to ABCB1	4.67 ± 0.45 ^r^###	0.28 ± 0.12
*ABCB1* (*Abcb1a* + *b1b*)	36.62 ± 54.00	295.34 ± 26.58 ^h^***; ^r^**	93.78 ± 47.90
*ABCB2*/*TAP1*	19.84 ± 19.30	14.98 ± 1.72	18.94 ± 5.33
*ABCB3*/*TAP2*	6.99 ± 8.53	47.64 ± 4.68 ^r^*; ^h^*	12.64 ± 20.52
*ABCB4*	0.84 ± 0.33	1.71 ± 0.04	3.21 ± 1.00 ^h^**
*ABCB5*	0.08 ± 0.03	0.00 ± 0.00	0.00 ± 0.00
*ABCB6*	19.99 ± 7.05	10.93 ± 1.61	17.39 ± 2.98
*ABCB7*	1.72 ± 0.41	9.95 ± 1.09 ^h^**; ^r^*	5.79 ± 2.54 ^h^*
*ABCB8*	7.08 ± 3.07	33.10 ± 9.46 ^h^**	40.43 ± 5.68 ^h^**
*ABCB9*	5.26 ± 2.20	21.47 ± 4.52 ^h^**	17.09 ± 4.28 ^h^*
*ABCB10*	1.88 ± 1.37	16.11 ± 1.24 ^h^**	14.00 ± 2.80 ^h^**
*ABCB11*	0.31 ± 0.25	0.14 ± 0.21	0.03 ± 0.01
*ABCC1*	0.90 ± 0.80	7.35 ± 0.19 ^h^**	6.52 ± 4.65 ^h^*
*ABCC2*	0.18 ± 0.11 ^m^*	0.03 ± 0.01	0.35 ± 0.05 ^m^***; ^h^**
*ABCC3*	0.41 ± 0.37	1.21 ± 0.59 ^h^*	0.98 ± 0.34
*ABCC4*	1.00 ± 1.02	77.02 ± 25.83 ^h^**; ^r^*	26.29 ± 6.88
*ABCC5*	10.00 ± 5.01	146.28 ± 22.30 ^h^****; ^r^****	17.69 ± 7.92
*ABCC6*	1.04 ± 0.74	15.72 ± 4.27 ^h^**; ^r^*	4.69 ± 3.00
*ABCC7*	0.13 ± 0.07	0.14 ± 0.02	0.04 ± 0.04
*ABCC8*	43.31 ± 37.99	14.67 ± 2.43	4.95 ± 1.34
*ABCC9*	2.40 ± 1.95	26.48 ± 10.26 ^h^**; ^r^*	8.14 ± 2.74
*ABCC10*	10.30 ± 4.29 ^r^*	5.89 ± 0.27	3.48 ± 1.13
*ABCC11*	0.23 ± 0.10	0.00 ± 0.00	0.00 ± 0.00
*ABCC12*	0.24 ± 0.20	1.99 ± 0.39 ^h^***; ^r^***	0.11 ± 0.02
*ABCC13*	0.01 ± 0.01	0.00 ± 0.00	0.00 ± 0.00
*ABCD1*	2.19 ± 1.04	12.84 ± 1.51 ^h^***; ^r^**	9.39 ± 2.51
*ABCD2*	0.53 ± 0.11	11.41 ± 1.92 ^h^**	11.61 ± 3.44 ^h^**
*ABCD3*	4.49 ± 1.70	39.19 ± 4.07 ^h^****	49.20 ± 3.48 ^h^****; ^m^*
*ABCD4*	21.42 ± 11.18	18.38 ± 1.36	6.57 ± 0.60
*ABCE1*	4.30 ± 1.77	33.30 ± 0.94 ^h^****; ^r^*	22.97 ± 4.96 ^h^***
*ABCF1*	8.61 ± 3.62	60.72 ± 1.79 ^h^****	63.48 ± 5.19 ^h^****
*ABCF2*	5.54 ± 2.98	54.18 ± 6.08 ^h^**	55.27 ± 21.99 ^h^**
*ABCF3*	19.77 ± 9.53	63.67 ± 4.50 ^h^***; ^r^*	42.97 ± 2.88 ^h^**
*ABCG1*	3.57 ± 2.96	29.25 ± 5.94 ^h^**	23.23 ± 6.08 ^h^**
*ABCG2*	10.01 ± 12.63	185.35 ± 38.42 ^h^**; ^r^*	83.22 ± 46.64
*Abcg3*	Not present	2.24 ± 1.14	18.27 ± 12.99
*ABCG4*	1.98 ± 1.49	30.69 ± 7.74 ^h^**; ^r^*	12.07 ± 4.17
*ABCG5*	0.08 ± 0.10	0.02 ± 0.02	0.42 ± 0.05 ^h^**; ^m^***
*ABCG8*	0.15 ± 0.08 ^m^*; ^r^*	0.01 ± 0.02	0.01 ± 0.02

^§^ One-way ANOVA: * *p* < 0.05; ** *p* < 0.01; *** *p* < 0.001; **** *p* < 0.0001; ^h^: compare to human; ^m^: compare to mouse; ^r^: compare to rat. § Two-tailed *t*-test: ## *p* < 0.01; ### *p* < 0.001.

**Table 2:** The levels of *ABC* transporter gene expression in human brain vessels and lung tissues (RNA-seq analysis by transcripts per million counts (TPM)) *.

ABC Transporter Genes	Human Brain Vessels (Mean ± SD)	Human Lung Tissues (Mean ± SD)
*ABCA1*	0.69 ± 0.66	2.40 ± 0.32 #
*ABCA2*	208.09 ± 70.27 ##	12.32 ± 5.17
*ABCA3*	10.85 ± 6.45	95.24 ± 49.02 #
*ABCA4*	0.19 ± 0.15	0.75 ± 0.56
*ABCA5*	4.51 ± 1.34	5.50 ± 0.23
*ABCA6*	0.88 ± 0.08	6.12 ± 1.13 ##
*ABCA7*	13.04 ± 3.68	19.18 ± 6.67
*ABCA8*	4.84 ± 1.54	6.8 ± 0.46
*ABCA9*	0.81 ± 0.91	1.24 ± 0.22
*ABCA10*	1.93 ± 1.26	3.17 ± 2.47
*ABCA12*	0.01 ± 0.00	0.03 ± 0.04
*ABCA13*	0.02 ± 0.01	0.54 ± 0.67
*ABCB1*	36.62 ± 54.00	4.35 ± 2.84
*ABCB2*/*TAP1*	19.84 ± 19.30	48.58 ± 16.21
*ABCB3*/*TAP2*	6.99 ± 8.53	20.72 ± 4.66
*ABCB4*	0.84 ± 0.33	0.38 ± 0.35
*ABCB5*	0.08 ± 0.03	0.08 ± 0.02
*ABCB6*	19.99 ± 7.05	7.10 ± 5.68
*ABCB7*	1.72 ± 0.41	6.94 ± 1.81 ##
*ABCB8*	7.08 ± 3.07	11.87 ± 1.11
*ABCB9*	5.26 ± 2.20 #	1.70 ± 0.30
*ABCB10*	1.88 ± 1.37	4.42 ± 2.15
*ABCB11*	0.31 ± 0.25	0.10 ± 0.04
*ABCC1*	0.90 ± 0.80	7.28 ± 1.60 ##
*ABCC2*	0.18 ± 0.11	0.32 ± 0.20
*ABCC3*	0.41 ± 0.37	21.07 ± 3.14 ###
*ABCC4*	1.00 ± 1.02	4.44 ± 2.86
*ABCC5*	10.00 ± 5.01	5.91 ± 2.24
*ABCC6*	1.04 ± 0.74	13.82 ± 1.98 ###
*ABCC7*	0.13 ± 0.07	8.30 ± 6.06
*ABCC8*	43.31 ± 37.99	1.78 ± 1.56
*ABCC9*	2.40 ± 1.95	2.74 ± 0.16
*ABCC10*	10.30 ± 4.29	9.85 ± 3.57
*ABCC11*	0.23 ± 0.10	0.06 ± 0.07
*ABCC12*	0.24 ± 0.20	0.03 ± 0.03
*ABCC13*	0.01 ± 0.01	0.00 ± 0.00
*ABCD1*	2.19 ± 1.04	3.95 ± 0.33 #
*ABCD2*	0.53 ± 0.11 ##	0.14 ± 0.07
*ABCD3*	4.49 ± 1.70	10.13 ± 3.67
*ABCD4*	21.42 ± 11.18	24.41 ± 4.06
*ABCE1*	4.30 ± 1.77	11.34 ± 4.15
*ABCF1*	8.61 ± 3.62	21.40 ± 3.42 #
*ABCF2*	5.54 ± 2.98	6.56 ± 0.97
*ABCF3*	19.77 ± 9.53	28.26 ± 2.61
*ABCG1*	3.57 ± 2.96	31.01 ± 16.02 #
*ABCG2*	10.01 ± 12.63	0.03 ± 0.02
*ABCG4*	1.98 ± 1.49	0.04 ± 0.03
*ABCG5*	0.08 ± 0.10	0.02 ± 0.01
*ABCG8*	0.15 ± 0.08	0.00 ± 0.00

* Two-tailed *t*-test: # *p* < 0.05; ## *p* < 0.01; ### *p* < 0.001.

**Table 3:** The levels of *ABC* transporter gene expression in mouse brain vessels vs. peripheral vessels and tissues (RNA-seq analysis by transcripts per million counts (TPM)) ^#^.

ABC Transporter Genes	Brain Vessels (Mean ± SD)	Liver Tissue (Mean ± SD)	Lung Vessels (Mean ± SD)	Spleen Tissues (Mean ± SD)
*Abca1*	19.45 ± 1.72 ^LT, LV, ST^**	4.29 ± 4.03	7.18 ± 3.59	5.02 ± 2.74
*Abca2*	107.57 ± 15.42 ^LT, LV, ST^***	2.02 ± 1.40	15.62 ± 5.47	4.81 ± 2.73
*Abca3*	35.98 ± 2.22 ^LT, ST^***	3.04 ± 3.04	37.80 ± 8.16 ^LT, ST^***	7.90 ± 4.63
*Abca4*	8.22 ± 1.19 ^LT, LV, ST^****	0.19 ± 0.27	2.54 ± 0.63 ^LT, ST^*	0.14 ± 0.09
*Abca5*	9.77 ± 1.16 ^LT, ST^**^; LV^*	0.32 ± 0.21	5.08 ± 2.65 ^LT, ST^*	0.29 ± 0.11
*Abca6*	0.67 ± 0.10	18.76 ± 9.39 ^BV, LV, ST^**	0.76 ± 0.24	0.16 ± 0.14
*Abca7*	19.29 ± 1.78 ^LT, ST^**	0.27 ± 0.12	24.35 ± 7.14 ^LT, ST^***	4.22 ± 1.23
*Abca8*	13.82 ± 3.03	14.47 ± 5.09	82.39 ± 16.70 ^BV, LT, ST^****	3.44 ± 0.96
*Abca9*	7.12 ± 1.23 ^LT^****^; LV, ST^***	0.24 ± 0.19	1.47 ± 0.46	1.70 ± 1.16
*Abca10*	0.00 ± 0.00	0.00 ± 0.00	0.00 ± 0.00	0.00 ± 0.00
*Abca12*	0.03 ± 0.01	0.00 ± 0.00	0.02 ± 0.02	0.00 ± 0.00
*Abca13*	0.03 ± 0.01	0.00 ± 0.00	0.01 ± 0.02	0.01 ± 0.01
*Abca14*	0.00 ± 0.01	0.06 ± 0.04	2.02 ± 1.30 ^LT, ST^*	0.03 ± 0.03
*Abca15*	0.00 ± 0.00	0.00 ± 0.00	0.00 ± 0.00	0.00 ± 0.00
*Abca16*	0.00 ± 0.00	0.00 ± 0. 00	0.01 ± 0.02	0.00 ± 0.00
*Abca17*	0.20 ± 0.13	0.02 ± 0.04	0.81 ± 0.44	0.10 ± 0.08
*Abcb1a*	290.67 ± 26.70 ^LT, LV, ST^****	0.27 ± 0.07	26.81 ± 7.34	3.37 ± 0.99
*Abcb1b*	4.67 ± 0.45 ^LT^***	0.80 ± 0.13	3.39 ± 1.12 ^LT^*	2.85 ± 0.81 ^LT^*
*Abca1a + b1b*	295.34 ± 26.58 ^LT, LV, ST^****	1.06 ± 0.09	30.19 ± 7.63	6.21 ± 0.13
*Abcb2*/*Tap1*	14.98 ± 1.72	4.12 ± 1.59	81.16 ± 24.53 ^BV, LT^**	58.37 ± 19.49 ^BV, LT^*
*Abcb3*/*Tap2*	47.64 ± 4.68 ^LT^**	4.00 ± 2.28	123.39 ± 20.66 ^LT^****^; BV, ST^***	58.10 ± 8.97 ^LT^**
*Abcb4*	1.71 ± 0.04	26.94 ± 18.47 ^BV, LV^*	0.45 ± 0.13	4.10 ± 0.87
*Abcb5*	0.00 ± 0.00	0.00 ± 0.00	0.00 ± 0.00	0.00 ± 0.00
*Abcb6*	10.93 ± 1.61	34.06 ± 9.07 ^BV, LV, ST^****	20.95 ± 2.10 ^BV^***^; ST^****	6.76 ± 0.87
*Abcb7*	9.95 ± 1.09 ^LT, LV, ST^**	3.46 ± 2.07	3.94 ± 0.81	3.99 ± 0.61
*Abcb8*	33.10 ± 9.46 ^ST^*	12.75 ± 2.54	28.88 ± 13.28 ^ST^*	6.89 ± 1.83
*Abcb9*	21.47 ± 4.52 ^LT, ST^****^; LV^***	0.42 ± 0.20	5.32 ± 0.57	3.24 ± 1.17
*Abcb10*	16.11 ± 1.24	12.51 ± 4.56	15.02 ± 1.10	9.96 ± 1.91
*Abcb11*	0.14 ± 0.21	89.50 ± 23.92 ^BV, LV, ST^****	0.28 ± 0.48	0.36 ± 0.53
*Abcc1*	7.35 ± 0.19 ^LT^***	0.19 ± 0.10	9.54 ± 1.78 ^LT^***^; ST^**	4.00 ± 2.05 ^LT^*
*Abcc2*	0.03 ± 0.01	15.76 ± 13.17	0.43 ± 0.18	0.06 ± 0.09
*Abcc3*	1.21 ± 0.59	16.62 ± 8.84 ^BV^*	29.72 ± 4.05 ^BV^***^; LT^*^; ST^**	11.97 ± 0.21
*Abcc4*	77.02 ± 25.83 ^LT, LV, ST^***	0.36 ± 0.27	2.60 ± 0.97	2.76 ± 1.38
*Abcc5*	146.28 ± 22.30 ^LT, ST^****^; LV^*	1.10 ± 0.54	96.94 ± 25.04 ^LT, ST^***	5.96 ± 1.90
*Abcc6*	15.72 ± 4.27 ^LV, ST^***	10.60 ± 3.70 ^LV, ST^**	0.19 ± 0.17	0.05 ± 0.07
*Abcc7*	0.14 ± 0.02	0.02 ± 0.00	0.25 ± 0.13 ^LT, ST^*	0.02 ± 0.02
*Abcc8*	14.67 ± 2.43 ^LT, LV, ST^****	0.05 ± 0.09	1.02 ± 0.28	0.05 ± 0.07
*Abcc9*	26.48 ± 10.26 ^LT, LV^**	1.03 ± 0.72	1.02 ± 0.40	2.07 ± 1.19
*Abcc10*	5.89 ± 0.27 ^LT, ST^**	0.36 ± 0.17	4.64 ± 2.13 ^LT, ST^**	0.65 ± 0.19
*Abcc11*	0.00 ± 0.00	0.00 ± 0.00	0.00 ± 0.00	0.00 ± 0.00
*Abcc12*	1.99 ± 0.39 ^LT, LV, ST^****	0.06 ± 0.04	0.20 ± 0.17	0.01 ± 0.01
*Abcc13*	0.00 ± 0.00	0.00 ± 0.00	0.00 ± 0.00	0.00 ± 0.00
*Abcd1*	12.84 ± 1.51	3.18 ± 2.39	26.75 ± 2.48 ^BV, ST^*^; LT^***	17.06 ± 6.80
*Abcd2*	11.41 ± 1.92 ^LT, ST^***^; LV^**	2.70 ± 2.12	4.23 ± 0.74	1.01 ± 0.12
*Abcd3*	39.19 ± 4.07 ^LV^*^; ST^****	100.43 ± 65.85 ^ST^*	26.40 ± 4.68 ^ST^***	4.73 ± 1.73
*Abcd4*	18.38 ± 1.36 ^LT, ST^**	2.05 ± 0.63	19.81 ± 6.34 ^LT, ST^***	1.86 ± 0.28
*Abce1*	33.30 ± 0.94 ^LT, ST^**	13.44 ± 6.11	26.15 ± 5.40 ^LT^*	16.58 ± 3.04
*Abcf1*	60.72 ± 1.79 ^LT^****^; ST^**	8.65 ± 4.83	65.19 ± 7.33 ^LT^****^; ST^***	34.83 ± 4.98 ^LT^**
*Abcf2*	54.18 ± 6.08 ^LT^****^; LV^**^; ST^***	9.84 ± 4.21	30.66 ± 3.54 ^LT^**	22.49 ± 3.93 ^LT^*
*Abcf3*	63.67 ± 4.50 ^LT, ST^****	16.19 ± 5.45	59.31 ± 9.07 ^LT^****^; ST^***	19.70 ± 0.78
*Abcg1*	29.25 ± 5.94 ^LT^**	0.80 ± 0.45	27.65 ± 2.70 ^LT^**	19.93 ± 9.74 ^LT^*
*Abcg2*	185.35 ± 38.42 ^LT, LV, ST^****	28.53 ± 15.63	24.84 ± 5.87	9.38 ± 1.22
*Abcg3*	2.24 ± 1.14	0.84 ± 0.21	9.07 ± 1.59 ^LT^*	25.01 ± 5.93 ^BV, LT^****^; LV^**
*Abcg4*	30.69 ± 7.74 ^LT, LV, ST^****	0.33 ± 0.35	0.45 ± 0.23	1.64 ± 0.34
*Abcg5*	0.02 ± 0.02	13.96 ± 8.20 ^LT, LV, ST^*	0.15 ± 0.19	0.09 ± 0.10
*Abcg8*	0.01 ± 0.02	10.87± 8.46	0.01 ± 0.02	0.12 ± 0.14

# One-way ANOVA: * *p* < 0.05; ** *p* < 0.01; *** *p* < 0.001; **** *p* < 0.0001; BV: compare to brain vessels; LT: compare to liver tissues; LV: compare to lung vessels; ST: compare to spleen tissues.

**Table 4:** The levels of *ABC* transporter gene expression in rat brain vessels vs. peripheral vessels and tissues (RNA-seq analysis by transcripts per million counts (TPM)) #.

ABC Transporter Genes	Brain Vessels (Mean ± SD)	Liver Tissues (Mean ± SD)	Lung Vessels (Mean ± SD)	Spleen Tissues (Mean ± SD)
*Abca1*	5.90 ± 1.47	11.81 ± 2.86	13.32 ± 6.25 ^ST^*	3.92 ± 0.90
*Abca2*	71.63 ± 25.12 ^LT^***^; LV, ST^**	4.19 ± 1.92	16.56 ± 5.44	5.97 ± 0.30
*Abca3*	26.49 ± 7.73 ^LT, ST^*	9.58 ± 2.57	113.33 ± 6.40 ^BV, LT, ST^****	9.57 ± 0.29
*Abca4*	7.85 ± 4.64 ^LT, LV, ST^*	0.20 ± 0.04	0.36 ± 0.08	0.97 ± 0.21
*Abca5*	2.60 ± 0.72 ^LT^*	0.54 ± 0.05	7.89 ± 2.98 ^LT, ST^**	0.11 ± 0.03
*Abca6*	0.03 ± 0.01	21.79 ± 4.20 ^BV, LV, ST^****	0.45 ± 0.07	0.03 ± 0.01
*Abca7*	9.86 ± 4.96	0.30 ± 0.11	19.32 ± 7.94 ^LT^**^; ST^*	5.72 ± 0.17
*Abca8*	13.52 ± 2.10	21.57 ± 2.51 ^ST^**	21.59 ± 9.15 ^ST^**	3.28 ± 0.50
*Abca9*	0.78 ± 0.26	0.48 ± 0.25	0.24 ± 0.18	0.50 ± 0.07
*Abca10*	0.00 ± 0.00	0.00 ± 0.00	0.00 ± 0.00	0.00 ± 0.00
*Abca12*	0.05 ± 0.02	0.00 ± 0.00	0.00 ± 0.01	0.00 ± 0.00
*Abca13*	0.05 ± 0.02	0.00 ± 0.00	0.00 ± 0.01	0.05 ± 0.01
*Abca14*	0.00 ± 0.00	0.01 ± 0.02	0.00 ± 0.00	0.00 ± 0.00
*Abca15*	0.00 ± 0.00	0.00 ± 0.00	0.00± 0.00	0.00 ± 0.00
*Abca16*	0.04 ± 0.04	0.69 ± 0.33 ^BV, LV, ST^**	0.03 ± 0.04	0.04 ± 0.01
*Abca17*	0.04 ± 0.02	0.07 ± 0.04	2.12 ± 1.04 ^BV, LT, ST^**	0.06 ± 0.01
*Abcb1a*	93.50 ± 47.78 ^LT, LV, ST^**	4.18 ± 1.07	2.43 ± 0.90	0.21 ± 0.08
*Abcb1b*	0.28 ± 0.12	1.97 ± 1.39	9.37 ± 7.72	0.42 ± 0.05
*Abca1a + b1b*	93.78 ± 47.9078 ^LT, ST^**^; LV^*	6.15 ± 2.46	11.80 ± 8.56	0.63 ± 0.13
*Abcb2*/*Tap1*	18.94 ± 5.33	11.65 ± 3.24	140.59 ± 55.46 ^BV, LV^**^; ST^*	53.59 ± 8.14
*Abcb3*/*Tap2*	12.64 ± 20.52	11.07 ± 4.69	61.88 ± 101.10	50.28 ± 3.97
*Abcb4*	3.21 ± 1.00	28.83 ± 16.53 ^BV, LV^*	3.62 ± 3.79	15.81 ± 2.31
*Abcb5*	0.00 ± 0.00	0.00 ± 0.00	0.00 ± 0.00	0.00 ± 0.00
*Abcb6*	17.39 ± 2.98	20.74 ± 3.91	20.01 ± 3.67	14.43 ± 1.16
*Abcb7*	5.79 ± 2.54	13.74 ± 1.09 ^BV^**^; LV^***^; ST^*	3.01 ± 0.45	9.19 ± 1.38 ^LV^**
*Abcb8*	40.43 ± 5.68 ^LT, ST^***^; LV^*	9.37 ± 4.18	25.33 ± 5.35 ^LT^**^; ST^*	9.66 ± 0.57
*Abcb9*	17.09 ± 4.28 ^LT, LV, ST^***	1.54 ± 0.77	2.47 ± 0.41	2.77 ± 0.54
*Abcb10*	14.00 ± 2.80	5.84 ± 2.34	9.59 ± 2.38	58.53 ± 6.4328 ^BV, LT, LV^***
*Abcb11*	0.03 ± 0.01	103.10 ± 6.5828 ^BV, LV, ST^****	0.33 ± 0.50	0.59 ± 0.13
*Abcc1*	6.52 ± 4.65	0.35 ± 0.20	3.50 ± 1.47	5.72 ± 0.75
*Abcc2*	0.35 ± 0.05	72.54 ± 23.86 ^BV, LV, ST^***	0.18 ± 0.02	0.06 ± 0.03
*Abcc3*	0.98 ± 0.34	2.16 ± 0.56	7.17 ± 1.76 ^BV, LT, ST^***	1.31 ± 0.02
*Abcc4*	26.29 ± 6.88 ^LT, ST^***^; LV^**	3.05 ± 1.41	7.92 ± 3.01	3.64 ± 0.70
*Abcc5*	17.69 ± 7.92 ^LT^**^; ST^*	0.40 ± 0.07	33.37 ± 24.38	5.19 ± 0.12
*Abcc6*	4.69 ± 3.00	21.87 ± 1.95 ^BV, LV, ST^****	3.36 ± 2.22	0.08 ± 0.01
*Abcc7*	0.04 ± 0.04	0.06 ± 0.06	1.20 ± 0.46 ^BV, LV, ST^**	0.01 ± 0.01
*Abcc8*	4.95 ± 1.34 ^LT^**^; LV^***	0.51 ± 0.12	0.28 ± 0.04	0.00 ± 0.00
*Abcc9*	8.14 ± 2.74 ^ST^*	4.05 ± 1.50	4.53 ± 3.51	1.47 ± 0.16
*Abcc10*	3.48 ± 1.13	0.84 ± 0.47	5.80 ± 1.60 ^LT^**	3.37 ± 0.16
*Abcc11*	0.00 ± 0.00	0.00 ± 0.00	0.00 ± 0.00	0.00 ± 0.00
*Abcc12*	0.11 ± 0.02 ^LT, LV, ST^***	0.01 ± 0.01	0.01 ± 0.02	0.01 ± 0.01
*Abcc13*	0.00 ± 0.00	0.00 ± 0.00	0.00 ± 0.00	0.00 ± 0.00
*Abcd1*	9.39 ± 2.51	18.47 ± 8.20	17.72 ± 6.65	11.54 ± 0.22
*Abcd2*	11.61 ± 3.44 ^LT, ST^**	2.64 ± 0.20	20.21 ±25.38	0.58 ± 0.06
*Abcd3*	49.20 ± 3.48 ^ST^**	93.03 ± 14.82 ^BV^**^; LV^***^; ST^****	44.92 ± 8.65 ^ST^**	6.19 ± 0.64
*Abcd4*	6.57 ± 0.60	1.28 ± 0.57	12.17 ± 4.39 ^LT, ST^**	2.18 ± 0.46
*Abce1*	22.97 ± 4.96	23.71 ± 0.63	19.48 ± 3.47	22.87 ± 3.26
*Abcf1*	63.48 ± 5.19 ^LT^**^; ST^*	25.22 ± 8.45	60.05 ± 13.24 ^LT^**	40.27 ± 4.01
*Abcf2*	55.27 ± 21.99 ^LT^*	19.43 ± 3.85	39.85 ± 12.28	23.77 ± 1.47
*Abcf3*	43.97 ± 2.88 ^LT^*^; ST^**	23.21 ± 6.41	39.58 ± 9.53 ^LT^*^; ST^*	19.44 ± 1.53
*Abcg1*	23.23 ± 6.08 ^LT^*	3.68 ± 1.21	299.66 ± 357.98	36.56 ± 3.12 ^BV^*^; LT^***
*Abcg2*	83.22 ± 46.64 ^LT, LV, ST^*	13.83 ± 2.39	1.17 ± 0.45	6.75 ± 0.97
*Abcg3*	18.27 ± 12.99	62.98 ± 15.33 ^BV^**	105.58 ± 126.24	35.68 ± 2.39
*Abcg4*	12.07 ± 4.17 ^LT^**^; LV^*	0.17 ± 0.04	3.34 ± 3.29	5.28 ± 0.97
*Abcg5*	0.42 ± 0.05	7.38 ± 3.68 ^BV, LV, ST^**	0.04 ± 0.01	0.03 ± 0.01
*Abcg8*	0.01 ± 0.02	2.36 ± 2.06	0.00 ± 0.01	0.02 ± 0.00

^#^ One-way ANOVA: * *p* < 0.05; ** *p* < 0.01; *** *p* < 0.001; **** *p* < 0.0001; BV: compare to brain vessels; LT: compare to liver tissues; LV: compare to lung vessels; ST: compare to spleen tissues.

## Data Availability

The data presented in this study are available upon request.

## Supplementary Materials

The following supporting information can be downloaded at https://www.mdpi.com/article/10.3390/pharmaceutics15051563/s1: Figure S1: Wes^TM^ analysis of ABCB1, ABCC1 and ABCG2 expression in human, mouse and rat brain vessels at the protein level; Table S1: ABC transporters and their functions.
