# Supplementary material for: Differential Expression of ABC Transporter Genes in Brain Vessels vs. Peripheral Tissues and Vessels from Human, Mouse and Rat
Source: Pharmaceutics. 2023 May 22;15(5):1563. doi: 10.3390/pharmaceutics15051563 (PMC10221359; doi:10.3390/pharmaceutics15051563)
Supplement: Supplementary file 1 [file pharmaceutics-15-01563-s001.zip › pharmaceutics-2332121-supplementary.pdf]

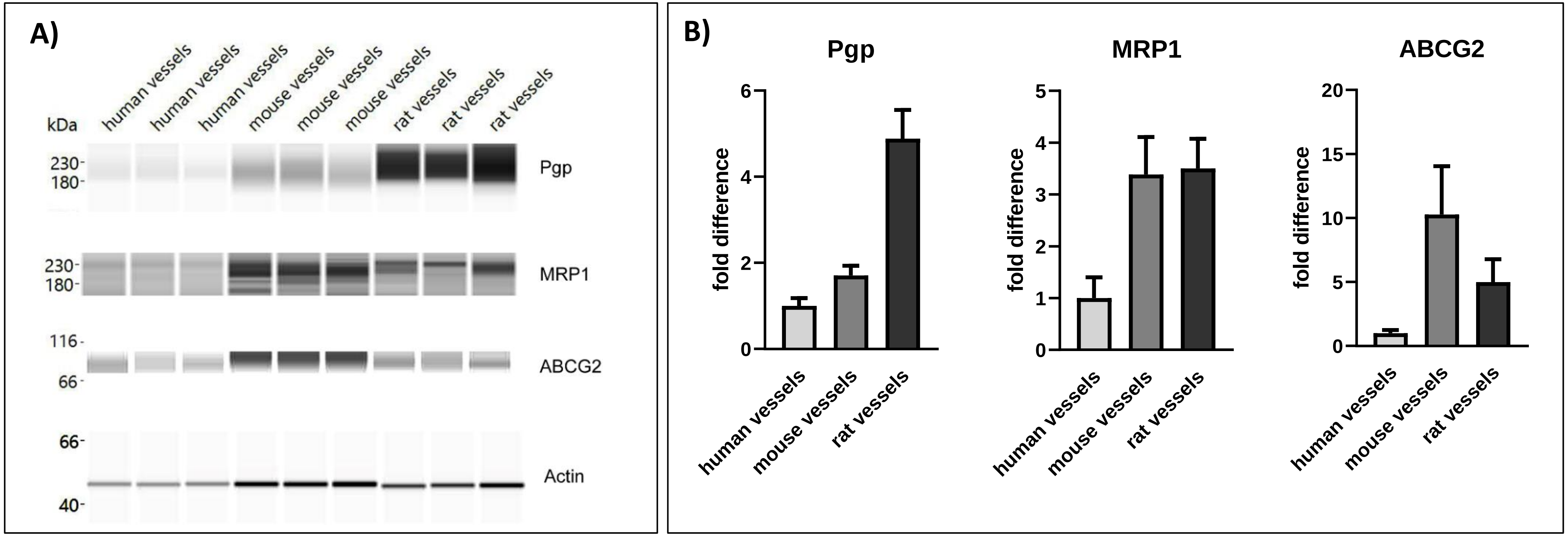

Supplementary Figure S1: Wes analysis of ABC transporter expression. **A)** Wes^{TM} analysis of ABCB1/Pgp, ABCC1/MRP1, and ABCG2 expression at the protein levels in cerebral vessels of human, mouse and rat. Three isolated cerebral vascular samples were used in the analysis from each of human, mouse and rats. **B)**. Densitometry analysis of ABCB1/Pgp, ABCC1/MRP1, and ABCG2 expression at the protein levels normalized against β-actin.

Supplementary Table S1. ABC transporters and their functions*.

Symbol	Functions and Diseases
ABCA1	Lipid transporter (Ch, PL); Regulate HDL metabolism; Lipidate ApoE; Modulate the production and deposition of Aβ; Tangier disease; FHDLD; AD; CAD§.
ABCA2	Transport of steroids, lipids and related molecules; Transport of SM (sphingomyelin), myelin; *ABCA2* knockout mice display developmental defects similar to aberrant myelination; Drug resistance to estramustine, estradiol, mitoxantrone and a free radical inithiator,2,2’-azobis-(2- amidinopropane) (AAPH); AD; Cancer.
ABCA3	Lipid transporter (PC, PG); Surfactant secretion; Newborn surfactant deficiency or RDS; expression in brain tissue.
ABCA4	N-retinylidiene-PE efflux; ABCA4 mutations are associated with retinal degenerations (Stargardt disease, retinitis pigmentosa, macular dystrophy); expression in CEC.
ABCA5	Intracellular sterol/steroid trafficking; ABCA5 is a protein related to lysosomal disease.
ABCA6	Cholesterol-response regulation; May play a role in macrophage lipid homeostasis
ABCA7	Lipid transporter (Ch, PL), Efflux of cellular cholesterol/phospholipids to HDL; expansion of the cellular ceramide; associated with Sjogren's syndrome; expression in brain tissue
ABCA8	The function of this protein has not yet been determined. May transport LTC4 (leukotriene C4)
ABCA9	Cholesterol-response regulation; macrophage lipid homeostasis? Transcriptional expression of this gene is induced during monocyte differentiation into macrophages and is suppressed by cholesterol import.
ABCA10	Cholesterol-response regulation; macrophage lipid homeostasis? Neither the substrate nor the function of this gene is known
ABCA12	ABCA12 mutation is associated with lamellar ichthyosis.
ABCA13	Rare variations and mutations in the gene may be linked to psychiatric disorders such as schizophrenia, bipolar disorder, and depression
ABCB1	Multidrug resistance/BBB; transport of drugs, lipid, sterol, peptides, cytokines; tissue defense; organ regeneration; SP phenotype; CAA (Cerebral Amyloid Angiopathy); AD; tumor; Parkinson
ABCB2	Peptide transport (peptide antigen presentation/immune response)
ABCB3	Peptide transport (peptide antigen presentation/immune response)
ABCB4	Phosphatidycholine and cholesterol transport; Cholestasis; ICP*; PFIC (progressive familial intrahepatic cholestasis).
ABCB5	Drug resistance to doxorubicin.
ABCB6	Fe/S cluster transport (mitochondria)
ABCB7	Fe/Sulphur cluster transport (sideroblastic anemia and ataxia) (mitochondria); may play a role in metal homeostasis.
ABCB8	Transport of heme, peptides and phospholipids. It may involve the compartmentalization and transport of heme, as well as peptides, from the mitochondria to the nucleus and cytosol. This protein may also play a role in the transport of phospholipids into mitochondrial membranes

ABCB9	Associated with lysosomal markers; peptide transport. May be involved in antigen presentation and in the translocation of peptides from the cytosol into the lysosomal lumen.
ABCB10	Heme transport (mitochondria)
ABCB11	Bile acid transport; ABCB11 mutations are found in PFIC2 patients.
ABCC1	Multidrug resistance/blood-CSF barrier; GS-X pump; transport of glutathione-conjugated drugs (eg LTC_4), GS-prostaglandin A_2, glucuronide conjugates (eg $E_2 17\beta G$), sulfate conjugates; peroxidation products, herbicides, mycotoxins, heavy metals, natural products, antifolate anti-cancer agents.
ABCC2	Transport of glucuronosyl derivatives; bilirubin, bile salt, organic anion (D-J syndrome*); GS- X pump; drug resistance to Vinblastine, methotrexate; transport of angiotensin receptor antagonists (valsartan, olmesartan)
ABCC3	GS-X pump; Drug resistance; Transport of glucuronidated compounds and methotrexate in liver and intestine; Transport of $E_2 17\beta G$, LTC_4, GS-DNP and organic anions.
ABCC4	GS-X pump; Nucleoside transport, Transport organic anionic compounds, sulfate conjugates and bile salt, resistance to nucleoside analogues (anti-viral drugs), antibiotics and antitumour agents; expression in CEC.
ABCC5	GS-X pump; Nucleoside transport, resistance to nucleoside analogues (anti-viral drugs). Transport organic anionic compounds, sulfate conjugates, heme, cAMP and cGMP, glutamate conjugate and analogs.
ABCC6	GS-X pump; Transport of glutathione conjugates and peptides. Mutations are associated with pseudoxanthoma elasticum. Transport GS-conjugates (e.g. LTC_4, GS-DNP)
ABCC7	Chloride channel; mutations result in cystic fibrosis.
ABCC8	ATP-sensitive K+ channel and insulin release; Sulfornylurea receptor; expressed in pancreatic insulin secreting cells and pituitary glands; FPHHI*
ABCC9	ATP-sensitive K+ channel
ABCC10	GS-X pump; Transport of estradiol (2)-17beta-glucuronide and; Drug resistance to paclitaxel. Transport LTC_4.
ABCC11	GS-X pump; Transport of bile acids, steroid sulfates (neurosteroids) in neuronal axons, cyclic nucleotides. Nucleoside and anticancer nucleotide analogues (eg. PMEA, 5-FU) transport; Transport a variety of lipophilic anions, including cyclic nucleotides, glutathione conjugates such as leukotriene C4 (LTC_4) and S-(2,4-dinitrophenyl)-glutathione (GS-DNP), steroid sulfates such as oestrone 3-sulfate (E1S) and dehydroepiandrosterone 3-sulfate (DHAES), glucuronides such as $E_2 17\beta G$, monoanionic bile acids glycocholate and taurocholate, and folic acid and its analogue methotrexate (MTX).
ABCC12	GS-X pump; Overexpressed in breast cancer.
ABCC13	This gene is now thought to be a pseudogene incapable of encoding a functional ABC protein.
ABCD1	Half-transporter; Peroxisomal membrane protein; Involved in peroxisomal import of fatty acids and/or fatty acyl-CoAs in the organelle; peroxisomal transport or catabolism of long chain fatty acids; Gene detect causes ALD
ABCD2	Half-transporter; Involved in peroxisomal import of fatty acids and/or fatty acyl-CoAs in the organelle; this protein is speculated to function as a dimerization partner of ABCD1 and/or other peroxisomal ABC transporters; late-onset cerebellar and sensory ataxia in Abcd2 knockout mice.

ABCD3	Half-transporter; require a partner half transporter molecule to form a functional homodimeric or heterodimeric transporter; May be involved in peroxisomal biogenesis
ABCD4	Half-transporter; May *function* as a heterodimer for another peroxisomal ABC transporter and, may modify the ALD phenotype. It may also play a *role* in the process of peroxisome biogenesis.
ABCE1	RNase L inhibitor; important in HIV-1 capsid assembly; implicated in tumor cell proliferation and antiapoptosis; required for both eukaryotic translation initiation as well as ribosome biogenesis.
ABCF1	This protein lacks the transmembrane domains; may be regulated by tumor necrosis factor-alpha and play a role in enhancement of protein synthesis and the inflammation process
ABCF2	Localized in mitochondrial membrane and cytoplasm. Acts as a suppressor of the volume-sensitive outwardly rectifying Cl channel; Overexpressed in ovary adenocarcinoma.
ABCF3	Localized in cytoplasm. Positively regulates cell proliferation, at least partially through the interaction with a tumor protein D52 protein family member TPD52L2. Display antiviral effect against flaviviruses.
ABCG1	Cholesterol/phospolipid transport; AD.
ABCG2	Toxin efflux; Multidrug resistance/blood-brain barrier; sterol transport; PC (phosphatidylcholine), PS (phosphatidylserine) transport; peptide transport; porphyrins and heme transport; regulate survival under low oxygen conditions; tissue defense; organ regeneration; SP (side population) phenotype; AD; breast cancer
Abcg3	Rodent; predicted to transport drugs; transport of peptides and hydrophobic compounds? High levels of expression in the thymus and spleen suggest a potential role in the transport of specific peptides or hydrophobic compounds from lymphocytes
ABCG4	Cholesterol/phospholipid transport.
ABCG5	Sterol transport
ABCG8	Sterol transport, Phytosterols, Shellfish transport.

* The functions of ABC transporters in this table are summarized based on Dean M et al. *Hum Mutat*. 2022, 43, 1162-1182; Gomez-Zepeda D, et al. ABC Transporters at the Blood-Brain Interfaces, Their Study Models, and Drug Delivery Implications in Gliomas. Pharmaceutics. 2019,12(1), 20; Zhang W et al. 2015. Expression and Function of ABC Transporters at the Blood-Brain Barrier. In. *The Blood-Brain Barrier in Health and Disease (Volume one)*, pp. 172-214. Ed. K. Dorovini-Zis, CRC press/Taylor & Francis Group, New York; Shen, S & Zhang, W. ABC transporters and drug efflux at the blood-brain barrier. *Rev Neurosci.* 2010, 21, 29-53.

§Abbreviations: ALD, adrenoleukodystrophy; CEC, cerebromicrovascular endothelial cells; D-J syndrome, Dubin-Johnson syndrome; FHDLD, familial hypoapoproteinemia; FPHHI, familial persistent hyperinsulinemic hypoglycemia of infancy; HDL, high-density lipoprotein; ICP, intrahepatic cholestasis of pregnancy; ND, not determined; PFIC2, Progressive familial intrahepatic cholestasis type 2.
AD (Alzheimer’s disease); CAD (coronary artery disease); Ch, cholesterol; PL, phospholipids; HDL, high density lipoprotein
PC, phosphatidylcholine; PG, phosphatidylglycerol; RDS, Respiratory disease syndrome.
